# Levetiracetam‐induced aggression and acute behavioral changes: A case report and literature review

**DOI:** 10.1002/ccr3.5586

**Published:** 2022-03-15

**Authors:** Jeff F Zhang, Ravi Piryani, Anil K Swayampakula, Osman Farooq

**Affiliations:** ^1^ Jacobs School of Medicine and Biomedical Sciences University at Buffalo Buffalo New York USA; ^2^ Division of Critical Care Medicine Department of Pediatrics University at Buffalo Buffalo New York USA; ^3^ Division of Pediatric Neurology Oishei Children's Hospital Buffalo New York USA

**Keywords:** antiepileptic drugs, behavior, child, epilepsy, Levetiracetam

## Abstract

Levetiracetam is a second‐generation antiepileptic medication used to treat a wide range of partial and generalized seizure disorders. While Levetiracetam is generally well‐tolerated, mild mood‐related side effects (e.g., anxiety, agitation, and depression) have been observed in a minority of patients in the days following initiation of therapy or changes in dosing. The development of acute aggression requiring termination of Levetiracetam therapy has been rarely reported in the medical literature but poses a limiting effect on treatment options for refractory epilepsy in pediatric patients. In this report, we present a teenage male patient with a history of seizure disorder who developed sudden, severe behavioral abnormalities and aggression following increases in his Levetiracetam dosing. His symptoms resolved rapidly after return of his medication dosing to baseline, with no further sequelae noted. Our observations suggest that Levetiracetam remains a safe and effective first‐line antiepileptic whose adverse behavioral side effect profile can be properly managed with close patient monitoring and dose titration.

## Abstract 2

While behavioral and mood‐related side effects have been observed in patients following initiation of or changes in their Levetiracetam dosing, we believe the benefits that Levetiracetam therapy offers in treating refractory epilepsy far outweighs any adverse effects, which can be managed adequately with close clinical monitoring and dose titration.

## 1 INTRODUCTION

Epilepsy is a common neurological disorder affecting children, with rates of incidence as high as 5.5 per 1000 in developed countries and 44 per 1000 in underdeveloped countries.^1^ Childhood epilepsy is also frequently comorbid with cognitive and developmental delays and the development of both psychiatric and behavioral disorders, including autism spectrum and attention‐deficit hyperactivity disorders. In a case‐control retrospective study examining this relationship, Anita et al^2^. reported finding the prevalence of behavioral comorbidities in pediatric patients to be significantly higher in epileptics compared with nonepileptics (39.1% vs. 7.9%, *p* < 0.001). This connection between epilepsy and behavioral disorders has been so widely reported in the medical literature that it has been referred to as the “Bidirectional Hypothesis,” which posits that the two conditions are manifestations of the same underlying functional impairments in common pathways responsible for regulating mood and behavior.^3^ Due to this increased susceptibility, extra care must be taken in the management of juvenile epileptic patients, particularly in the utilization of antiepileptic medications that carry a risk of adverse behavioral side effects. While Levetiracetam therapy is generally well‐tolerated in pediatric patients, mood‐related changes, including agitation, depression, and anxiety, are infrequent side effects that have been reported secondary to initiation of therapy, though they are generally tolerable and mild in presentation.^4^ In this case report, we present a 13‐year‐old Caucasian boy with a history of unspecified seizure disorder well‐managed on his home dosing of Levetiracetam and Clobazam who suddenly developed severe aggressive behavioral changes following increases in his Levetiracetam dose during a short hospitalization course for status epilepticus.

## 2 CASE PRESENTATION

The patient was a 13‐year‐old Caucasian boy, born prematurely at 25 weeks, with a medical history of seizure disorder requiring vagal nerve stimulator (VNS) placement, interventricular hemorrhage status post ventriculoperitoneal shunt placement, congenital deafness, legal blindness, and cerebral palsy, who presented to the emergency department with status epilepticus in the setting of febrile illness. His antiepileptic medication regimen consists of 600 mg of Levetiracetam twice a day and 10 mg of Clobazam once daily with no recent changes in medications or missed doses. The patient typically has seizures roughly twice a month, which last between 30 and 60 s in duration. On this evening, he had seizures intermittently for 2 h with whole body stiffening, facial twitching, and nystagmus. His mother insisted that the patient had been acting normally prior to the onset of seizures and is normally calm and cooperative with her at baseline. She denies any sick contacts, recent trauma, or missing vaccinations. Activation of his vagal nerve stimulator was unsuccessful in terminating the seizures. Emergency medical services were called, and the patient was taken to the emergency department.

In the emergency department, the patient was found to be actively seizing. He was administered a total of 9 mg of Lorazepam, 2000 mg of Levetiracetam and 15 ml of Acetaminophen IV, after which his seizures were terminated. Laboratory results were only significant for leukocytosis (WBC) of 12.6 × 10^9^/L with prominent neutrophilia. He was empirically started on meningitic doses of Vancomycin and Ceftriaxone and transferred to the pediatric intensive care unit. Further evaluation of the patient's ventriculoperitoneal shunt was unremarkable. His Levetiracetam dose was increased from 600 mg BID to 950 mg BID in addition to receiving a loading dose of 950 mg of Levetiracetam IV for improved seizure prophylaxis. Over the next 24 h, the patient began to display significant agitation, including multiple episodes of screaming, crying, thrashing, and attempts to self‐harm (e.g., head banging and throwing himself on the floor). The patient was found to be extremely aggressive towards hospital staff and his mother requiring multiple doses of sedatives. It was suspected that the increase in Levetiracetam dosing was the reason for his acute agitation, and the dose was reduced to his preadmission dose for the remainder of his hospital stay. The patient's behavior became noticeably and rapidly calmer over the next 2 days. No clinical or electrographic seizures were noted throughout the patient's hospital course, nor was there any recurrence of fever since admission. Blood and cerebrospinal fluids showed no growth. Following improvements in the patient's behavior to baseline and negative 72‐h culture results, empiric antibiotics were terminated and the patient was discharged home on Day 4 postadmission.

## 3 DISCUSSION

Levetiracetam is a second‐generation antiepileptic used to treat a range of partial and generalized seizures, and juvenile myoclonic epilepsy.^5^ While its precise mechanism of action is unclear, it is thought that Levetiracetam exerts its antiepileptic effects by binding to and inhibiting synaptic vesicle protein SV2A, thereby decreasing the rate of presynaptic neurotransmitter release and increasing the seizure threshold.^6^ While Levetiracetam therapy demonstrates a number of pharmacokinetic advantages compared with other antiepileptic drugs (including absence of drug–drug interactions and reduced cytochrome P450 enzyme induction due to its partial extrahepatic metabolism),^5^ it also has a number of side effects associated with initiation of treatment or acute changes in dosage. The most common adverse effects in adults are the development of somnolence, weakness, and dizziness; while in children, its behavioral adverse effects are more notable.^5^ Phase III studies of the drug initially reported that more than 13% of a mixed group of both pediatric and adult patients were noted to have secondary symptoms of agitation, hostility, anxiety, depression, depersonalization, and emotional lability associated with use.^7^ A systematic review of pediatric patients alone conducted by Halma et al.^8^ correspondingly found that 62 out of 203 (30.1%) patients developed behavioral side effects secondary to Levetiracetam treatment, with the most common symptoms being hostility, aggression, and anxiety (30 of 62 symptomatic patients, 48.4%). White et al^9^. reported in a case series of 553 patients that 38 of them (6.9%) required discontinuation of Levetiracetam therapy soon after treatment initiation due to the development of severe, intolerable behavioral side effects. Most notably, their study also found that the patients in the discontinuation group had been administered a significantly lower dose of Levetiracetam compared to the patients who were able to continue treatment,^9^ suggesting that the development of adverse behavioral effects is likely associated with some variable degree of susceptibility reflective of the underlying pathologic changes in neural circuitry resulting from a particular patient's chronic seizure disorder.

It is thought that dysregulation of neural circuits resulting from excitotoxic damage in patients with epilepsy subsequently decreases their “cerebral reserve” for resisting the adverse effects of Levetiracetam therapy, thus predisposing them to rapid decompensation when exposed to increased doses of the drug.^9^ While the pathophysiology behind Levetiracetam's effects on behavior is uncertain, there are so far no reports in the medical literature of studies demonstrating toxic effects on neurons following exposure to the drug. On the contrary, animal studies have shown that Levetiracetam exerts neuroprotective properties in cases of stroke and traumatic brain injury through its ability to modify calcium channels and prevent further ischemic neuronal apoptosis.^10^ Perhaps more informative of Levetiracetam's pathologic mechanism of action are pharmacodynamic studies on the structurally related drug Brivaracetam, which has 15–30 times the affinity to the SV2A protein as Levetiracetam.^4^ However, treatment of epileptic patients with Brivaracetam has been associated with a significantly lower incidence of behavioral changes compared with Levetiracetam.^4^ Despite their structural similarity, Brivaracetam notably does not share the latter's negative modulatory effect on NMDA receptors,^4^ suggesting Levetiracetam might induce psychiatric disturbance and aggressive behavior in patients in a manner similar to Phencyclidine, another NMDA receptor antagonist.

The clinical relevance of side effects caused by Levetiracetam is significant in that the mood and behavioral intolerability of the drug for patients and their caregivers limits the extent to which the drug is able to be utilized in the pediatric population. Fortunately, it has been observed that few patients stop Levetiracetam therapy even after the development of these types of side effects, suggesting that they are either mild or do not present a significant change from baseline behavior.^11^ Bertsche et al. found that Levetiracetam monotherapy in pediatric patients was much more likely to result in treatment failure (defined as a “lack of effectiveness”) compared with Valproate or Oxcarbazepine monotherapies (Levetiracetam vs. Valproate vs. Oxcarbazepine: 41% vs. 22% vs. 29%, *p* ≤ 0.05), while less than 7% (4 of 61 patients) of patients treated with Levetiracetam discontinued therapy due to the development of behavioral and mood disorders alone.^11^ In addition, as in the case of our patient, multiple other case studies^12^, ^13^, ^14^ have reported that the adverse effects of Levetiracetam are transient and resolve rapidly following discontinuation of therapy with a complete return to baseline functioning. These findings suggest that Levetiracetam remains an effective first‐line antiepileptic, despite the possibility of adverse behavioral side effects as, with careful monitoring, the risks of initiating therapy are low and benefits high in being able to successfully treat refractory epilepsy in pediatric patients.

## 4 CONCLUSIONS

Side effects related to Levetiracetam therapy initiation or dosing changes frequently present as mild behavioral or mood‐related changes, but the onset of acute aggression has been rarely reported in the literature. This case report documents a patient who developed sudden and severe behavioral changes following increases in his Levetiracetam dosing that resolved quickly following a return of his medication dosing to baseline, with no further sequelae. Our observations suggest that the risk of developing adverse behavioral reactions should not dissuade clinicians from utilizing Levetiracetam in cases of refractory epilepsy as, with careful monitoring and dose titration, it remains a safe and effective first‐line antiepileptic agent.

## AUTHOR CONTRIBUTIONS

Jeff F Zhang: drafted, edited, and approved the manuscript, accountable for manuscript integrity. Ravi Piryani: edited and approved the manuscript. Anil K Swayampakula and Osman Farooq: involved in project conception, edited the manuscript, and approved the manuscript.

## CONSENT

Written informed consent was obtained from the patient to publish this report in accordance with the journal's patient consent policy.

## Data Availability

Data sharing not applicable to this article as no datasets were generated or analyzed during the current study.
